# LncRNA MBNL1-AS1 Represses Proliferation and Cancer Stem-Like Properties of Breast Cancer through MBNL1-AS1/ZFP36/CENPA Axis

**DOI:** 10.1155/2022/9999343

**Published:** 2022-04-26

**Authors:** Yu Ding, Yingjie Li, Yunqiang Duan, Wan Wang, Wei Zheng, Weilun Cheng, Yuan Qi, Jianyuan Feng, Ziang Chen, Tianshui Yu, Anbang Hu, Ting Wang, Mingcui Li, Hanyu Zhang, Yanling Li, Fei Ma, Baoliang Guo

**Affiliations:** ^1^Department of General Surgery, The Second Affiliated Hospital of Harbin Medical University, Harbin, China; ^2^Department of Pathology, The Second Affiliated Hospital of Harbin Medical University, Harbin, China; ^3^Department of Breast Surgery, China-Japan Union Hospital of Jilin University, Changchun, China; ^4^Department of Endocrinology, The Second Affiliated Hospital of Harbin Medical University, Harbin, China

## Abstract

**Background:**

Emerging studies have revealed long noncoding RNAs (lncRNAs) were key regulators of cancer progression. In this research, the expression and roles of MBNL1-AS1 were explored in breast cancer (BC).

**Methods:**

In this study, the MBNL1-AS1 expression in breast cancer tissue, as well as in cell line, was studied by qRT-PCR assays. The effects of MBNL1-AS1 on proliferation and stemness were evaluated by MTT assays, colony formation assays, orthotopic breast tumor mice models, extreme limiting dilution analysis (ELDA), fluorescence in situ hybridization (FISH), flow cytometry assays, and sphere formation assays. Flexmap 3D assays were performed to show that MBNL1-AS1 downregulated the centromere protein A (*CENPA*) secretion in BC cells. Western blot, RNA pull-down assays, RNA immunoprecipitation (RIP) assays, and FISH were conducted to detect the mechanism.

**Results:**

The results showed that the expression levels of MBNL1-AS1 were downregulated in breast cancer tissues and cell lines. In vitro and in vivo studies demonstrated that overexpression of MBNL1-AS1 markedly inhibited BC cells proliferation and stemness. RNA pull-down assay, RIP assay, western blot assay, and qRT-PCR assay showed that MBNL1-AS1 downregulated *CENPA* mRNA via directly interacting with Zinc Finger Protein 36 (ZFP36) and subsequently decreased the stability of *CENPA* mRNA. Restoration assays also confirmed that MBNL1-AS1 suppressed the *CENPA*-mediated proliferation and stemness in breast cancer cells.

**Conclusions:**

The new mechanism of how MBNL1-AS1 regulates BC phenotype is elucidated, and the MBNL1-AS1/ZFP36/*CENPA* axis may be served as a therapeutic target for BC patients.

## 1. Introduction

Female breast cancer has surpassed lung cancer as the most common cancer, with about 2.3 million new cases (11.7%) [1]. It is the main cause of cancer-associated death for women around the world [2]. Approximately 15.4% of women died of breast cancer, although the diagnosis and treatment strategies improved greatly over the past several decades [1]. The abnormal proliferation of tumor cells is a striking feature of malignant tumors. Cancer stem cells (CSCs) have vital roles in intra- and intertumoral heterogeneity, which are related to tumor progression, treatment resistance, and disease relapse [3, 4]. Therefore, exploring the potential molecular mechanisms underlying breast cancer proliferation and stemness is of paramount importance in identifying effective and novel therapeutic strategies.

LncRNAs, classes of noncoding RNAs (ncRNAs), have greater than 200 nucleotides length [5, 6]. and were able to modulate several phenotypes such as proliferation, metastasis, stemness, and progression of cell cycle [7–10]. Accumulating researches had showed that the expression pattern of lncRNAs was correlated with cancer progression, proliferation, and stemness [10–13]. Emerging evidence demonstrated that lncRNAs regulated the expression level of a target gene by binding to RNA-binding proteins (RBPs). For instance, the lncRNAs interacted with the RBPs and subsequently regulated cancer progression [14, 15]. These researches showed that lncRNAs were pivotal players in cancer pathogenesis and vital new biomarkers in cancer early detection and therapy. Recently, two cohorts were analyzed to find lncRNAs of differential expression by research in BC. MBNL1-AS1 was regarded as one of the downregulated lncRNAs in BC [13]. However, the phenotype and mechanism of MBNL1-AS1 in BC have not been detected.

*CENPA* (a 17 kDa variant of histone H3) was located in the active centromeres [16, 17]. A previous study has found that *CENPA* was correlated with human pluripotent stem cell self-renewal [18]. Low expression of *CENPA* induced cell cycle arrest and promoted apoptosis [19]. Subsequent evidence showed that upregulation of *CENPA* promoted initiation and progression in several cancers [17, 20]. Higher expression of *CENPA* was correlated with increased invasiveness and higher-grade cancers [19, 21]. In contrast, downregulation of *CENPA* showed to inhibit the HCC cells proliferation [19]. Moreover, *CENPA* had predictive value in breast cancer and could contribute to disease progression as a marker of proliferation [21, 22]. In this research, MBNL1-AS1 was founded markedly decreased the expression of *CENPA* mRNA.

Here, the study showed that MBNL1-AS1 in BC was downregulated and the decreased expression of MBNL1-AS1 was correlated with survival. MBNL1-AS1 inhibited BC proliferation, and stem cell properties were confirmed by functional studies in vitro and in vivo. Mechanistically, MBNL1-AS1 directly interacted with ZFP36, an RNA-binding protein, subsequently reduced the stabilization of *CENPA* mRNA. This study might bring new insights into therapeutic targets for BC.

## 2. Materials and Methods

### 2.1. Cell Lines and Human BC Specimens

Human BC cell lines (MCF-7, MDA-MB-468, and MDA-MB-231) and the immortalized normal breast cell line MCF-10A were purchased from the Cell Bank of Chinese Academy of Sciences (Shanghai, China). Indicated cells were cultured in DMEM containing 10% FBS (Thermo Fisher Scientific) and were supplied at 37°C in a humidified atmosphere of 5% CO_2_. 60 BC samples were obtained from the Department of Breast Surgery, The Second Affiliated Hospital of Harbin Medical University (246 Xuefu Street, Nangang District, Harbin, China). The 60 patients were Asian women, aged from 33 to 77 years. Fresh BC tissues and paired adjacent normal tissues were frozen in liquid nitrogen immediately after surgical excision and saved at -80°C. The tissues were validated by H&E staining. This research was approved by the Ethic Committee of Harbin Medical University.

### 2.2. qRT-PCR Assay

To isolate total RNA, the TRIzol reagent (Life Technologies, Carlsbad, CA, USA) was performed. Next, DNAse Treatment was used to remove genomic DNA. It was performed as previously detailed described [23]. For cDNA synthesis, the total RNA was retrotranscribed with the PrimeScript® RT Reagent Kit (Takara). The SYBR Premix Ex Taq™ Kit (Takara, Tokyo, Japan) was utilized to carry out qPCR. And RT-PCR was conducted on a 7500 RealTime PCR System. GAPDH was measured as the internal control. The sequences of the primers that were used are as follows: for MBNL1-AS1: 5′- CTCCCGCTTCTTCTACCGAC -3′ (forward), 5′- TTGGTGCATTTTAAGGCGGC -3′ (reverse); for *CENPA*: 5′- GATTCTGCGATGCTGTCTG -3′ (forward), 5′- GCCTTTGGAACGGTGTT -3′ (reverse); for ZFP36: 5′- TCCACAACCCTAGCGAAGAC -3′ (forward), 5′- GAGAAGGCAGAGGGTGACAG -3′ (reverse); for GAPDH, 5′- CTCCTCCACCTTTGACGCTG -3′ (forward), 5′- TCCTCTTGTGCTCTTGCTGG -3′ (reverse). The primer sequences of qRT-PCR used in the study are listed in Table 1. All data were calculated by 2^−*ΔΔ*CT^ method.

### 2.3. Cell Transfection

To assess the MBNL1-AS1 overexpression vector, the lentiviral vector-MBNL1-AS1 and lentiviral vector-convirus (Genepharma, Shanghai, China) were transfected in cells. sh-MBNL1-AS1#1, sh-MBNL1-AS1#2, and sh-control were obtained from GenScript (Nanjing, China). The qRT-PCR assays were used to detect the expression of MBNL1-AS1. The sequences of the MBNL1-AS1 targeting shRNAs were as follows: for MBNL1-AS1-shRNA#1: 5′- GATCCGAACGAAAGGAGCAGGGTATTTCAAGAGAATACCCTGCTCCTTTCGTTTTTTTA-3′ (sense), 5′- AGCTTAAAAAAACGAAAGGAGCAGGGTATTCTCTTGAAATACCCTGCTCCTTTCGTTCG-3′ (antisense); for MBNL1-AS1-shRNA#2: 5′- GATCCGCCAGAACCTAGTCTCATGTTTCAAGAGAACATGAGACTAGGTTCTGGTTTTTA-3′ (sense), 5′- AGCTTAAAAACCAGAACCTAGTCTCATGTTCTCTTGAAACATGAGACTAGGTTCTGGCG-3′ (antisense); for NC-shRNA: 5′- GATCCCCTTCTCCGAACGTGTCACGTTTCAAGAGAACGTGACACGTTCGGAGAATTTTT -3′ (sense), 5′- AGCTAAAAATTCTCCGAACGTGTCACGTTCTCTTGAAACGTGACACGTTCGGAGAAGGG -3′ (antisense). siRNA, si-ZFP36, and si-NC were obtained from RiboBio (Shanghai, China). The sequences are listed in Table 2. Lipofectamine 2000 (Invitrogen) was used for the transfections of shRNAs and siRNAs in breast cancer cells. The cells were harvested 48 h after transfection for RNA extraction or functional assays.

### 2.4. MTT Study

Transfected cells were seeded in a 96-well plate (Corning Life Science) which had 3 × 10^3^ cells/well. Each well was added with 10 *μ*L MTT (5 mg/mL) for different periods of time. After 4 hours of incubation, 150 *μ*l of dimethyl sulfoxide was added to dissolve the precipitates. The absorbance was then measured by detection at 560 nm using a microplate reader. The experiment was performed independently in triplicate.

### 2.5. Colony Formation Analysis

The BC cells were harvested and plated in a 6-well plate (0.2 × 10^3^ cells/well) and cultured in incubators. After 14 days of culture, the plate was washed 3 times with PBS. The indicated BC cells were dried and stained with 0.1% crystal violet solution at 37°C for 15 minutes. Then, the cell colonies formed were numbered under a digital camera.

### 2.6. Sphere Formation Assay

A total of 5 × 10^3^ cells were plated in 6-well ultralow attachment surface plates (Corning Life Science). EGF (20 ng/ml, Invitrogen), bFGF (10 ng/ml, Invitrogen), and 2% B27 (Invitrogen, Carlsbad, CA) were formulated in a medium. After 2 weeks, the number of spheroids was counted manually by a microscope.

### 2.7. Extreme Limiting Dilution Assay

Extreme limiting dilution assay was performed as previously described [24]. Briefly, MCF-7 or MDA-MB-231 cells were plated in 96-well ultralow attachment surface plates at a density of 100, 250, 500, and 1000 cells/well, with 12 replicates for each cell density at 37°C in a humidified atmosphere of 5% CO_2_. The number of wells presenting visible tumor spheres was counted on after 14 days, and then the data was put into the ELDA online software (http://bioinf.wehi.edu.au/software/elda/). The stem cell frequency was calculated.

### 2.8. Flow Cytometry Assay

Flow cytometry assay was performed as previously described [25, 26]. Briefly, using flow cytometry with the use of propidium iodide (Sigma-Aldrich, MO China) staining (5 *μ*g/ml), cell cycle analysis was performed after transfection. The cells were trypsinized and dispersed into cell suspension. Next, 1 mL of cell suspension was centrifuged at 1,500 rpm for 10 min. Then, the indicated cells were added with chilled ethanol and saved at 4°C overnight. Then, the BC cells were centrifuged and resuspended in PBS and appended with 100 *μ*L RNase at room temperature. After 30 min, 100 *μ*l PI was appended, and cells were incubated at 37°C in dark for 60 minutes. Cell cycle was determined by FACScaliber Flow Cytometer. MCF-7 and MDA-MB-231 cells were labeled with FITC-conjugated CD44 antibody and PE-conjugated CD24 antibody at 4°C for 15 min. Data were analyzed with a FACS Aria II cell sorter (BD, Bioscience, USA).

### 2.9. Western Blot

Total protein of indicated cells was extracted using RIPA lysis buffer (Beyotime, Jiangsu, China) with protease inhibitors. Cell lysates were separated on 10% SDS-PAGE. A PVDF membrane was transferred by the indicated protein, which was blocked with 5% non-fat dry milk. Primary antibodies, namely, Ab against ZFP36 (1 : 1000), Ab against *CENPA* (1 : 1000), and Ab against GAPDH (1 : 1000) (all from Thermo Fisher Scientific, USA), were incubated with the membrane. Subsequently, the secondary antibodies (1 : 10000) (Thermo Fisher Scientific, USA) were incubated with the membrane for 1 h. After developed by enhanced chemiluminescent and exposure, immunoreactive protein band intensities were analyzed by Image J software.

### 2.10. RNA Pull-Down

The biotin-labeled MBNL1-AS1 plasmid and biotin-labeled antisense RNA plasmid were, respectively, transfected in BC cells. Biotin-labeled RNA was bound to the Streptavidin agarose Beads, next mixed with indicated cell lysates. Indicated bound RNAs were isolated from the beads by washed and boiled for normal western blot. The assays were experimented in RNase-free conditions.

### 2.11. RNA Immunoprecipitation (RIP) Assay

RIP was performed with the use of the Magna RIP kit (Millipore, USA). The spectrophotometer (Thermo Scientific, USA) was used to examine the RNA concentration, and the bio-analyzer (Agilent, USA) was utilized to detect the RNA quality. The input control was the total RNA. Following, qRT-PCR analysis was used to test the results to demonstrate the bound targets.

### 2.12. Magnetic Luminex® Performance Assay

Magnetic Luminex® performance assay was performed based on our previous study [14]. Briefly, NC or MBNL1-AS1 was transfected in MDA-MB-231 cells, which were incubated for 1 day. Following centrifugation, the supernatant was collected. Then, the following human cytokines: *PLK1*, *PAF*, *CENPA*, *YB-1*, *TWIST*, *YY1*, *KLF4*, *CUG2*, *E2F8*, *SALL4*, *RAE1*, and *PTPA* were analyzed by FlexMAP 3D (Luminex®) platform.

### 2.13. Immunofluorescence Staining (IF) and Fluorescence In Situ Hybridization (FISH) Assay

IF and FISH assays were performed as previously described [14, 26]. For FISH, the MBNL1-AS1 subcellular localization was assessed by the lncRNA FISH Probe Mix and FISH kit which was obtained from Guangzhou RiboBio Co., Ltd. The 4% paraformaldehyde-fixed cells were fixed in PBS. After washed by phosphate buffered solution with tween for three times, the anti-fluorescence quencher sealed the indicated cells, and the images were taken by the FV1000 laser microscope (Olympus, Japan). For IF, cells were fixed with 4% formaldehyde and subjected to standard immunofluorescence staining. The anti-CENPA and secondary antibodies were obtained from Thermo Fisher Scientific. The data was analyzed by Image J software. The nuclei were dyed with DAPI. The 3D-cultured spheroids were conducted as previously described [26].

### 2.14. mRNA Decay Assay

Stable cells were added with 5 *μ*g/mL actinomycin D. Following, qRT-PCR was performed to determine the *CENPA* mRNA. The data was performed independently in triplicate.

### 2.15. Animal Studies

Animal studies were ratified by the ethics committee of Harbin Medical University (Harbin, China). The BC cells were injected in the flank of four-week-old female nude mice (each group has 3 mice). The volume of tumor was recorded every week. After intraperitoneal injection of D-luciferin, all mice were imaged by the Xenogen IVIS Spectrum Imaging System (Caliper Life Sciences, USA) before being sacrificed. Primary tumor volume was measured. The mice were euthanized by i.p. injection of sodium pentobarbital (200 mg/kg).

### 2.16. Statistical Analysis and Database

All analyses were experimented three times at least. The measurement data in this study were exhibited as means ± SD. Student's *t*-test was applied to compare the numeric variables between two groups. The differences between more than two groups of variables were evaluated by *χ*^2^ or Fisher's exact *χ*^2^-test. R software package version 3.0.0 and GraphPad Prism 5 were used. The TCGA data set from the GEPIA2 Platform was used to analyze the expression of MBNL1-AS1 in tumor and normal tissues. And Kaplan-Meier Plotter was used to analyze the RFS in high and low expression of MBNL1-AS1. lncLocator database (http://www.csbio.sjtu.edu.cn/bioinf/lncLocator/) was used to predict the subcellar localization of MBNL1-AS1. A *p*-value<0.05 was regarded as significant; *p*-value <0.01 was very significant.

## 3. Results

### 3.1. MBNL1-AS1 Expression Was Downregulated in BC Cells Which Was Correlated with Poor Prognosis

**Figure 1:**
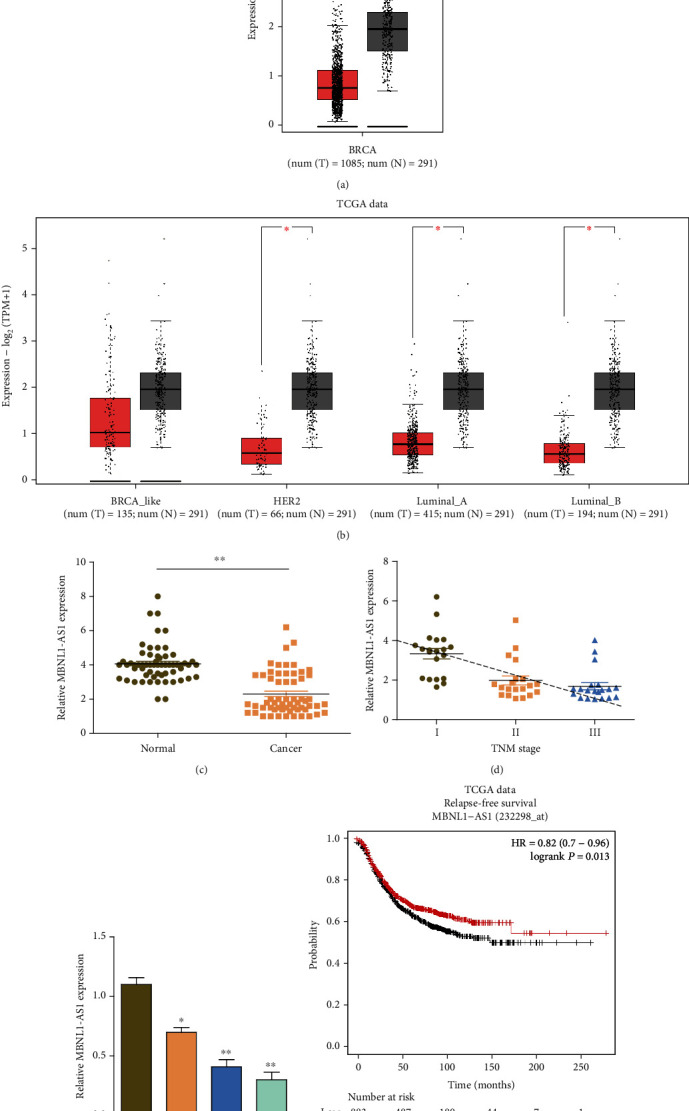
MBNL1-AS1 was decreased in breast cancer and correlated with survival. (a) The TCGA data set from the GEPIA2 Platform revealed that the expression levels of MBNL1-AS1 were downregulated in BC tissues compared with normal breast tissues. (b) The MBNL1-AS1 expression in different BC subtypes of TCGA data. (c) qRT-PCR revealed a markedly lower level of MBNL1-AS1 in our 60 BC patients. (d) The expression levels of MBNL1-AS1 were detected in different TNM stages of our 60 BC patients. (e) qRT-PCR analysis of MBNL1-AS1 pattern in MCF-10A, MCF-7, MDA-MB-468, and MDA-MB-234 cell lines. (f) Kaplan-Meier analysis indicated a better RFS in patients with high MBNL1-AS1 expression in a group of 1764 patients. Experiments were performed independently in triplicate. ∗*P* < 0.05, ∗∗*P* < 0.01.

MBNL1-AS1 was regarded as one of the LncRNA suppressor genes associated with breast cancer, according to the bioinformatics analysis [13]. However, expression and function in breast tissue have not been confirmed yet. Therefore, the MBNL1-AS1 expression was detected in the cancer genome atlas (TCGA) database. As indicated in Figure 1(a), the MBNL1-AS1 expression pattern was marked lower than normal tissues in BC tissues. The MBNL1-AS1 levels in different BC subtypes were explored for the following study. The MBNL1-AS1 levels in tumor tissues were found markedly decreased in HER2+, Luminal A, and Luminal B subtypes compared with normal tissues. However, no statistical significance of MBNL1-AS1 expression was found in basal-like subtype between tumor tissues and normal tissues (Figure 1(b)). Next, the MBNL1-AS1 expression level was examined in 60 BC tissues and normal breast tissues. The H&E staining of the BC patients and normal sample is shown in Supplementary Figure 1A. Through the qRT-PCR, MBNL1-AS1 expression significantly decreased in the BC tissues (Figure 1(c)). Then, we linked the expression of MBNL1-AS1 with the clinicopathological features of the BC patients. We observed that the expression of MBNL1-AS1 had negative relationship with TNM stage and lymph node metastasis (Figure 1(d) and Table 3). Then, we detected the MBNL1-AS1 expression in several BC cell lines. Compared with the immortal MCF-10A cells and low-metastatic BC cell line MCF-7, the MBNL1-AS1 expression levels were observed to be much lower in BC cell lines, particularly in MDA-MB-468 and MDA-MB-231 cell lines with the higher metastatic features (Figure 1(e)). Given this expression mode, MCF-7 and MDA-MB-231 cell lines were selected to perform the following functional study. The Kaplan-Meier Plotter was used to make clear the relationship between the expression levels of MBNL1-AS1 and the patients' survival. The samples were grouped into high or low MBNL1-AS1 expression by median. The results showed that lower MBNL1-AS1 expression was markedly correlated to worse relapse-free survival (RFS) (Figure 1(f)). Altogether, the results demonstrated that the downregulated MBNL1-AS1 in BC might associate with a poor prognosis.

### 3.2. MBNL1-AS1 Inhibited Proliferation and Stemness of BC In Vitro and In Vivo

**Figure 2:**
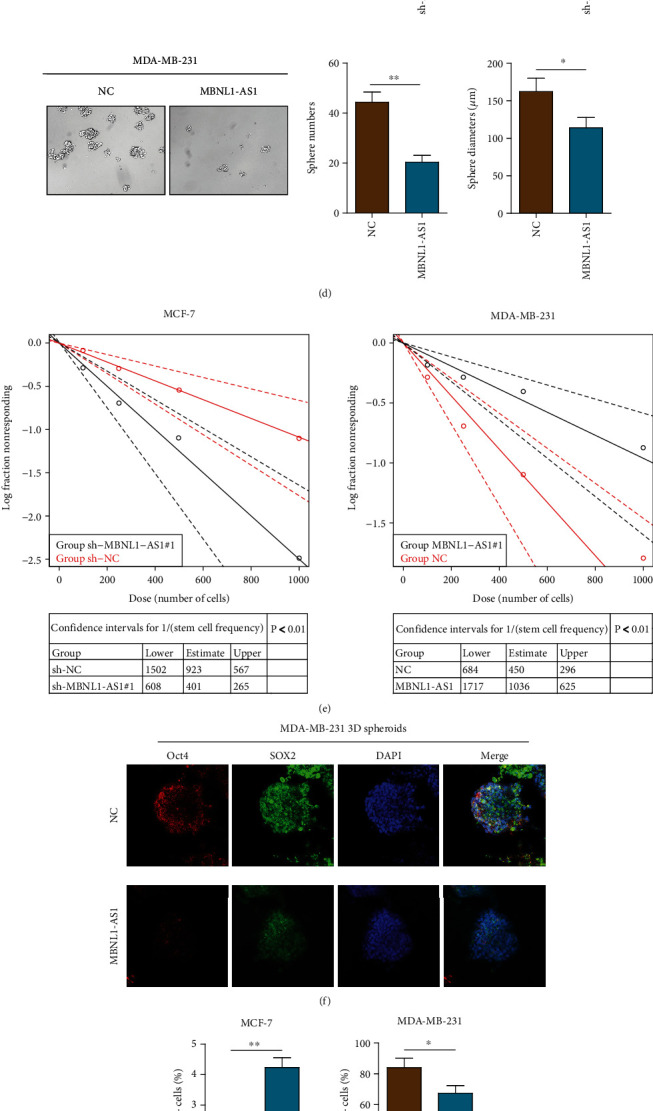
Biological functions of MBNL1-AS1 in BC. (a) qRT-PCR of MBNL1-AS1 in indicated cells transfected with shRNA-MBNL1-AS1#1/#2 (sh-MBNL1-AS1#1/#2) or shRNA-control (sh-NC) and MBNL1-AS1 or the scramble control sequence in MCF-7 cell lines and MDA-MB-231 cell lines. MTT assays (b) and colony formation assays (c) were used to examine the proliferation of MCF-7 cells with MBNL1-AS1 knockdown and MDA-MB-231 cells with MBNL1-AS1 overexpression. (d) Number and diameters of tumor spheres in indicated cells. (e) The extreme limiting dilution analysis (ELDA) for calculation of sphere initiating BC frequency in MCF-7 and MDA-MB-231 cells. (f) IF staining of stemness markers (Sox2 and Oct4) in control or MBNL1-AS1-transfected MDA-MB-231 cells. (g) The CD44+/CD24- phenotype was determined in MCF-7 and MDA-MB-231 cells transfected with sh-MBNL1-AS1 and MBNL1-AS1 by flow cytometry analysis. (h) The BC cells in different phases were detected by flow cytometry analysis. (i) Orthotopic breast tumor mice models were used to explore the growth of tumors. Tumor diameters were evaluated every week. The data was performed independently in triplicate.

The biological functions of MBNL1-AS1 in BC were examined by observing the relationship between expression of MBNL1-AS1 and BC prognosis. MBNL1-AS1 levels were silenced by shRNA in MCF-7 cells and overexpressed in MDA-MB-231 cells. RT-PCR analysis was also utilized to confirm the efficiency (Figure 2(a)). As shown in Figure 2(b), knockdown of MBNL1-AS1 by shRNA markedly enhanced the growth of MCF-7 cells. In contrast, MBNL1-AS1 over-expression significantly inhibited the proliferation abilities of MDA-MB-231 cells. Colony formation assays further confirmed the anti-proliferation of MBNL1-AS1 in the BC cells (Figure 2(c)). Next, the sphere formation assay was used to detect the stemness of BC cells. The stemness properties of MCF-7 cells increased significantly by knockdown of MBNL1-AS1, whereas the stemness properties of MDA-MB-231 cells decreased markedly as overexpression of MBNL1-AS1 (Figure 2(d)). To further verify the stem cell characteristics of MBNL1-AS1 in BC, ELDA, immunofluorescence staining, and flow cytometry assays were utilized to evaluate the stemness markers of BC cells (Figures 2(e)–2(g)). ELDA results showed that MBNL1-AS1 largely restrained the self-renew ability of the cells (Figure 2(e)). The expression of OCT4 and SOX2 decreased in MBNL1-AS1-transfected MDA-MB-231 cells (Figure 2(f)). Flow cytometry assay results showed that sh-MBNL1-AS1 transfection in MCF-7 cells increased the percentage of CD44+CD24− cells, whereas MBNL1-AS1 overexpression decreased the percentage of CD44+CD24− MDA-MB-231 cells (Figure 2(g)). The results of knocking down MBNL1-AS1 by sh-MBNL1-AS1#2 in functional studies are shown in Supplementary Figures 2A-2C. Flow cytometry assays confirmed that MBNL1-AS1 influenced the cell cycle, evidenced by the G0/G1- cells were increased and the S- and G2/M- cells were reduced (Figure 2(h)). To confirm the effect of MBNL1-AS1 in vivo, MCF-7 cells transfected with sh-MBNL1-AS1 showed dramatically increased tumorigenic abilities in vivo. Tumorigenic abilities were significantly inhibited in MBNL1-AS1 over-expression mice compared with control mice (Figure 2(i)). Collectively, the results revealed that MBNL1-AS1 suppressed the BC cells proliferation and stemness abilities.

### 3.3. MBNL1-AS1 Suppressed the Expression of *CENPA* by Reducing the Stability of *CENPA* mRNA

**Figure 3:**
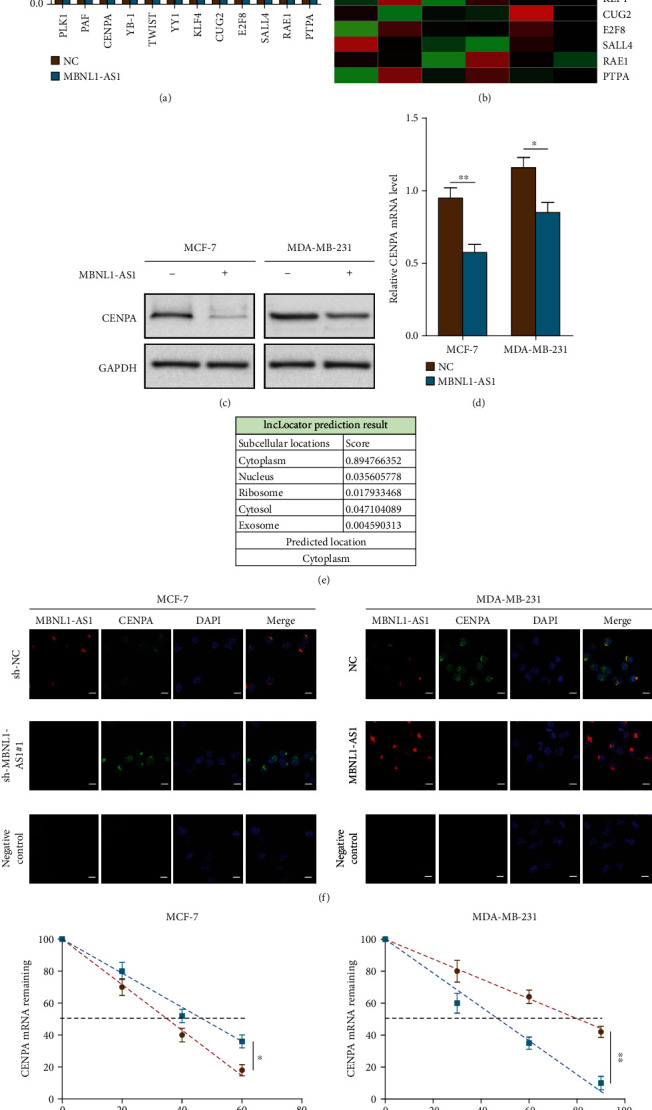
MBNL1-AS1 inhibited the expression of *CEPNA* by reducing the stability of *CENPA* mRNA. The proliferation- and stemness-associated factors were detected and shown by Flexmap liquichip assays (a) and the heat map (b). The expression levels of *CENPA* in the BC cells were tested by western blot assays (c) and qRT-PCR (d). (e) lncLocator database was used to predict the subcellular localization of MBNL1-AS1. (f) Subcellular localization in indicated cells was examined by combined IF/FISH assays. Scale bars =10 *μ*m. (g) The abundance of *CENPA* in indicated cells treated with actinomycin D was detected by qRT-PCR. Experiments were performed independently in triplicate. ∗*P* < 0.05, ∗∗*P* < 0.01.

To elucidate the mechanism which MBNL1-AS1 suppresses the proliferation and stemness of breast cancer cells, we analyzed some known factors closely related to the proliferation and stemness of BC by flexmap liquichip assays. The results showed that overexpression of MBNL1-AS1 in MDA-MB-231 cells significantly inhibited the *CENPA* secretion; however, other proteins were not of statistical significance (Figures 3(a) and 3(b)). We, therefore, set out to detect the regulatory effect of MBNL1-AS1 on *CENPA* in BC cells. Interestingly, MBNL1-AS1 significantly reduced the levels of *CENPA* protein and mRNA in BC cell lines (Figures 3(c) and 3(d)). Since the subcellular location of lncRNAs determined the functions, lncLocator database (http://www.csbio.sjtu.edu.cn/bioinf/lncLocator/) was used to predict subcellular localization of MBNL1-AS1 (Figure 3(e)). The result showed that MBNL1-AS1 localized mainly in cytoplasm. We further confirmed that by FISH assay (Figure 3(f)), implying that *CENPA* might be regulated in a post-transcriptional manner. As shown in Figure 3(f) and Supplementary Figure 2D, MBNL1-AS1 knockdown markedly upregulated the *CENPA* expression in MCF-7 cells and vice versa in other cell lines. We next detected whether or not MBNL1-AS1 regulated the *CENPA* mRNA in breast cancer cells. As expected, after the treatment of actinomycin D (an RNA synthesis inhibition agent), the *CENPA* mRNA half-life in MBNL1-AS1-silenced MCF-7 cells was dramatically prolonged while the *CENPA* mRNA half-life shortened after MBNL1-AS1 was transferred to MDA-MB-231 cells (Figure 3(g)). In conclusion, MBNL1-AS1 inhibited the *CEPNA* expression by reducing the stability of *CENPA* mRNA.

### 3.4. MBNL1-AS1 Directly Interacted with ZFP36 and Subsequently Reduced the Stability of *CENPA* mRNA

**Figure 4:**
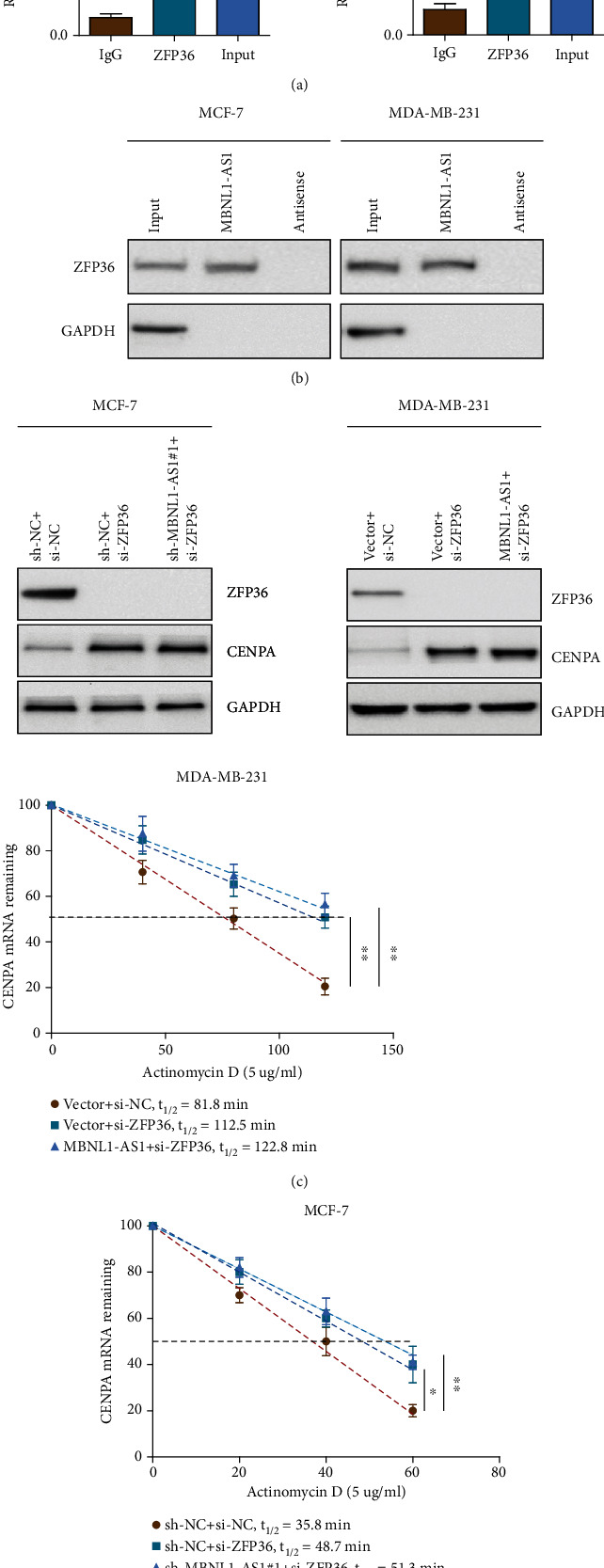
MBNL1-AS1 interacted with ZFP36 and reduced the *CENPA* mRNA stability. (a) RIP assays and (b) RNA pull-down assays were used to examine the combination of MBNL1-AS1 and ZFP36 in MCF-7 and MDA-MB-231 cells. (c) The expression of ZFP36 and *CENPA* were detected using western blot. (d) qRT-PCR assays were performed to examine the half-life of *CENPA* mRNA in the BC cells. The data was performed independently in triplicate.

To investigate how MBNL1-AS1 regulated *CENPA*, the RBPDB software was used to predict target proteins of MBNL1-AS1. ZFP36 was found that could directly bind with MBNL1-AS1. Besides, researches showed that ZFP36 can specifically bind to AU-rich elements (ARE) in the mRNA 3'UTR and subsequently induce mRNA decay [27, 28]. RIP assays were used to examine whether MBNL1-AS1 was physically associated with ZFP36. As indicated in Figure 4(a), these results demonstrated that MBNL1-AS1 could directly bind with ZFP36 in indicated cells. RNA pull-down analysis further demonstrated the interaction of MBNL1-AS1 and ZFP36. ZFP36 proteins were pulled down by MBNL1-AS1, but the antisense RNA was not pulled down (Figure 4(b)). To examine whether MBNL1-AS1 bound to ZFP36 and modulated the *CENPA* mRNA stability, we tested the *CENPA* mRNA half-life in indicated cells after ZFP36 was silenced. Before that, western blot assay was used to examine the ZFP36 knockdown efficiency in the BC cells (Figure 4(c)). As shown in Figures 4(c) and 4(d), MBNL1-AS1 could not affect the *CENPA* mRNA stability after ZFP36 silenced, nor decrease the protein pattern of *CENPA* in indicated cells. To conclude, MBNL1-AS1 directly interacted with ZFP36 and reduced the *CENPA* mRNA stability.

### 3.5. MBNL1-AS1 Suppressed Proliferation and Stemness of Breast Cancer Cells by Interacting with ZFP36

**Figure 5:**
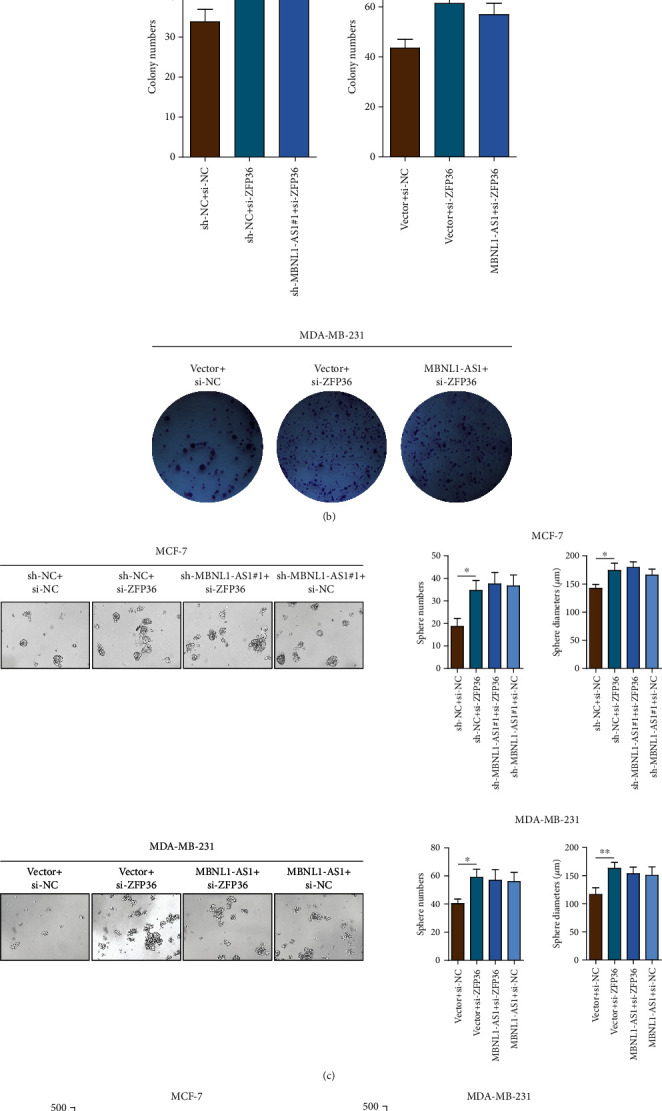
MBNL1-AS1 suppressed proliferation and stemness abilities of BC cells by interacting with ZFP36. MTT assay (a) and colony formation assay (b) were performed to measure the proliferation of MCF-7 cells and MDA-MB-231 cells. (c) Sphere formation assay was performed to analyze the stemness numbers and diameters of indicated cells. (d) Orthotopic breast tumor mice models were used to explore the growth of breast tumors. Tumor diameters were evaluated every week. (e) The MBNL1-AS1/ZFP36/*CENPA* axis regulated proliferation and stemness in BC cells and then affected BC progression. Experiments were performed independently in triplicate.

To further validate our findings, MTT assays, colony formation assays, and sphere formation assays were performed to examine the function of ZFP36 in breast cancer cell lines and xenograft tumor models. ZFP36 knockdown significantly enhanced the growth of MCF-7 cells; however, knockdown of MBNL1-AS1 did not revert the proliferation abilities (Figures 5(a) and 5(b)). The same results also showed that MBNL1-AS1 overexpression did not affect the growth of MDA-MB-231 cells after ZFP36 knockdown. Next, we examined the stemness abilities of indicated cells. As indicated in Figure 5(c), ZFP36 knockdown enhanced the stemness abilities of MCF-7 cells. However, knockdown of MBNL1-AS1 did not revert the stemness abilities of indicated cells. Similarly, sphere formation assays showed that MBNL1-AS1 knockdown or overexpression failed to revert the increase of sphere numbers in si-ZFP36 transfected BC cells. Meanwhile, the results of in vivo assays also confirmed that transfection of MBNL1-AS1 or si-MBNL1-AS1 did not revert the tumorigenic abilities after ZFP36 knockdown in orthotopic breast cancer mice models (Figure 5(d)). Our data revealed that the interaction of MBNL1-AS1 and ZFP36 inhibited the proliferation and stemness of BC cells.

## 4. Discussion

LncRNAs are known as diagnostic markers for several kinds of cancers including BC. Previous research demonstrated various lncRNAs as vital players in BC progression [13, 26]. In this research, the MBNL1-AS1 expression in breast cancer patients and its functions in BC cells were detected and investigated. The results indicated that expression of MBNL1-AS1 markedly decreased in breast cancer and high-metastatic BC cell lines, which was in line with the studies that revealed that MBNL1-AS1 levels were downregulated in colorectal cancer, NSCLC, retinoblastoma, and bladder cancer. Consistent with our research, another report showed that MBNL1-AS1 inhibited progression of NSCLC by sponging miR-135a-5p, which also identified MBNL1-AS1 as a tumor suppressor factor [29]. Also in NSCLC, a study revealed an inhibitory role of MBNL1-AS1 in CSC drug resistance of NSCLC by upregulating miR-301b-3p-targeted TGFBR2 [30]. Similarly, MBNL1-AS1 inhibited proliferation of bladder cancer through MBNL1-AS1/MiR-362-5p/QKI and MBNL1-AS1/miR-135a-5p/phlpp2/foxo1 signaling pathway [31, 32]. The similar mechanism occurs in colon cancer; the study revealed an inhibitory role of MBNL1-AS1 by upregulating miR-412-3p-targeted MYL9 [33]. MBNL1-AS1/miR-338-5p/wnt/*β*-catenin signaling pathway also showed the anti-tumor effect in retinoblastoma [34]. These strongly demonstrated that MBNL1-AS1 might be a reliable biological marker to diagnose BC. The clinical significance of MBNL1-AS1 still needs to be further verified in more samples for the limitations of the present study. Moreover, MBNL1-AS1 inhibited the proliferation and stemness of breast cancer cells in vitro and inhibited the tumorigenesis of breast cancer cells in vivo as demonstrated by these results of gain- or loss-of-function studies. These results indicated that MBNL1-AS1 played antioncogenic roles in BC.

Mounting evidence showed that lncRNAs regulated the downstream gene expression by binding to RBPs and competing endogenous RNAs (ceRNAs) [14, 26, 35, 36]. ZFP36 was known as RBP, which bound to the target mRNAs untranslated regions subsequently reduced their stability [35, 37–39]. It was studied that the ZFP36 expression was downregulated in BC and regulated the stability of *CENPA* mRNA [38]. However, the upstream regulating factors except ZFP36 are still being researched. Our study demonstrated that the ability of ZFP36 to modulate the stability of *CENPA* mRNA was regulated by MBNL1-AS1. One research reported that the AP1 transcriptional factor components, including JUN, could transcriptionally regulate the expression of ZFP36 [40]. To clear if MBNL1-AS1 regulates the expression of ZFP36, so, we conducted western blot assays. The results showed that MBNL1-AS1 failed to regulate ZFP36 expression Supplementary Figure 3A). The data could strengthen our hypothesis that MBNL1-AS1 interacts with ZFP36 and promotes the function of ZFP36 as an RNA-binding protein. MBNL1-AS1 directly interacted with ZFP36 and subsequently reduced the stabilization of *CENPA* mRNA. Similar regulatory mechanisms were also found in previous studies [35, 41]. In this research, the stability of *CENPA* mRNA was modulated by MBNL1-AS1 (Figure 3(a) and Figure 3(f)). Interestingly, in Figure 3(a), *PLK1*, *PAF*, *YB-1*, *TWIST*, *YY1*, *KLF4*, and *SALL4* expressions were decreased but with no statistical significance in MBNL1-AS1 transfected cells. We find that MBNL1-AS1 has been reported to inhibit the progression of prostate cancer by sponging miR-181a-5p and regulating PTEN/PI3K/AKT/mTOR signaling [42] and PTEN signaling happens to be regulated by *PLK1*, *PAF*, *YB-1*, *TWIST*, *YY1*, *KLF4*, and *SALL4* [43–48]. Therefore, *PLK1*, *PAF*, *YB-1*, *TWIST*, *YY1*, *KLF4*, and *SALL4* might be the indirect downstream targets of MBNL1-AS1. As for *CUG2*, *E2F8*, *RAE1*, and *PTPA*, they were reported to be the downstream targets of several other factors, like *NPM1*, *NONO*, *NKG2D*, and *UBE3A* [49–52]. So, *CUG2*, *E2F8*, *RAE1*, and *PTPA* might not be the downstream targets of MBNL1-AS1 in breast cancer cells. Therefore, they may remain unchained. However, the possibility of translation of the MBNL1-AS1 control *CENPA* mRNA has not yet been detected. Thus, further study is still needed to the development of how MBNL1-AS1 regulated the *CENPA* expression.

MBNL1-AS1 was regarded as one of the tumor suppressors of lncRNAs in BC [13]. Nevertheless, no studies had confirmed the MBNL1-AS1 expression pattern in BC. Therefore, the expression pattern of MBNL1-AS1 was detected for the first time in breast cancer tissues and cell lines. MBNL1-AS1 was confirmed that the expression in breast cancer and highly metastatic cells was downregulated. That result was consistent with the bioinformatic data from TCGA. In functional assays, an anti-stemness and anti-proliferation function of MBNL1-AS1 was shown by decreasing *CENPA* expression. In accordance with our study, the researchers also confirmed that MBNL1-AS1 was a tumor-suppressive lncRNA [29, 31–34]. We demonstrated that MBNL1-AS1 attenuated the abilities of breast cancer stemness and proliferation through reduced stability of *CENPA* mRNA, which unraveled a novel mechanism of MBNL1-AS1. Undoubtedly, the MBNL1-AS1 and the interplay network and the vital roles of MBNL1-AS1 in BC were enriched and verified by this study.

## 5. Conclusions

We demonstrated that MBNL1-AS1 levels were downregulated in BC tissues, which were correlated with prognosis. In vitro and in vivo assays unraveled the anti-stemness and anti-proliferation roles of MBNL1-AS1. In mechanism, MBNL1-AS1 interacted with ZFP36 and subsequently reduced the stabilization of *CENPA* mRNA (Figure 5(e)). Therefore, we elucidated a novel mechanism for how MBNL1-AS1 regulated the phenotype of BC and targeting the MBNL1-AS1/ZFP36/*CENPA* axis might function as therapeutic targets for breast cancer patients.

## Figures and Tables

**Table 1:** The primer sequences of qRT-PCR used in this study.

Name		Sequence (5′-3′)
MBNL1-AS1	Forward	CTCCCGCTTCTTCTACCGAC
	Reverse	TTGGTGCATTTTAAGGCGGC
ZFP36	Forward	TCCACAACCCTAGCGAAGAC
	Reverse	GAGAAGGCAGAGGGTGACAG
CENPA	Forward	GATTCTGCGATGCTGTCTG
	Reverse	GCCTTTGGAACGGTGTT
GAPDH	Forward	CTCCTCCACCTTTGACGCTG
	Reverse	TCCTCTTGTGCTCTTGCTGG

**Table 2:** The RNA sequences used in this study.

Name		Sequence (5′-3′)
sh-MBNL1-AS1#1	Sense	GATCCGAACGAAAGGAGCAGGGTATTTCA AGAGAATACCCTGCTCCTTTCGTTTTTTTA
	Antisense	AGCTTAAAAAAACGAAAGGAGCAGGGTAT TCTCTTGAAATACCCTGCTCCTTTCGTTCG
sh-MBNL1-AS1#2	Sense	GATCCGCCAGAACCTAGTCTCATGTTTCAA GAGAACATGAGACTAGGTTCTGGTTTTTA
	Antisense	AGCTTAAAAACCAGAACCTAGTCTCATGTT CTCTTGAAACATGAGACTAGGTTCTGGCG
NC-shRNA	Sense	GATCCCCTTCTCCGAACGTGTCACGTTTCA AGAGAACGTGACACGTTCGGAGAATTTTT
	Antisense	AGCTAAAAATTCTCCGAACGTGTCACGTTC TCTTGAAACGTGACACGTTCGGAGAAGGG
si-ZFP36	Sense	UGUGAAAGUGAUCCGCGAC
	Antisense	GUCGCGGAUCACUUUCACA
si-NC	Sense	GCCAUGUAUACGCGGUUC
	Antisense	GAACCGCGUAUACAUGGCC

**Table 3:** Relationship between MBNL1-AS1 expression and clinicopathologic features of BC patients (*n* =60).

Variable	Relative MBNL1-AS1 expression		*P*-value
	Low (*n* =30)	High (*n* =30)	
Age			NS
<50	16	13	
>50	14	17	
Histological differentiation			NS
Well	9	13	
Moderate	12	12	
Poor	9	5	
Tumor size			NS
<2 cm	8	17	
2-5 cm	11	6	
> 5 cm	11	7	
Lymph node metastasis			P<0.05
Yes	18	9	
No	12	21	
Tumor stage			P<0.05
I	6	16	
II	13	9	
III	11	5	

Note: BC patients were divided into MBNL1-AS1 high group and low group according to the analysis of qRT-PCR detection. NS: not significant between different groups. Differences among variables were evaluated by *χ*^2^ or Fisher's exact *χ*^2^ -test.

## Data Availability

The data used or analyzed during the current study are available from the corresponding author on reasonable request.

## Supplementary Materials

Supplementary Materials Supplementary Figure 1. The HE-stained tissues of BC and normal. Supplementary Figure 2. The function assays of MCF-7 cells transfected with sh-MBNL1-AS1#2. Supplementary Figure 3. The expression levels of ZFP36 in the BC cells.

## References

[1] Sung H., Ferlay J., Siegel R. L. (2021). Global cancer statistics 2020: GLOBOCAN estimates of incidence and mortality worldwide for 36 cancers in 185 countries. *CA: a Cancer Journal for Clinicians*.

[2] Siegel R. L., Miller K. D., Fuchs H. E., Jemal A. (2021). Cancer statistics, 2021. *Statistics*.

[3] Brooks M. D., Burness M. L., Wicha M. S. (2015). Therapeutic implications of cellular heterogeneity and plasticity in breast cancer. *Cell Stem Cell*.

[4] Visvader J. E., Lindeman G. J. (2012). Cancer stem cells: current status and evolving complexities. *Cell Stem Cell*.

[5] Cech T. R., Steitz J. A. (2014). The noncoding RNA revolution--trashing old rules to forge new ones. *Cell*.

[6] Guttman M., Russell P., Ingolia N. T., Weissman J. S., Lander E. S. (2013). Ribosome profiling provides evidence that large noncoding rnas do not encode proteins.

[7] Bhan A., Soleimani M., Mandal S. S. (2017). Long noncoding RNA and cancer: a new paradigm. *Cancer Research*.

[8] Mercer T. R., Dinger M. E., Mattick J. S. (2009). Long non-coding RNAs: insights into functions. *Nature Reviews Genetics*.

[9] Hu W. L., Jin L., Xu A. (2018). GUARDIN is a p53-responsive long non-coding RNA that is essential for genomic stability. *Nature Cell Biology*.

[10] Tang T., Guo C., Xia T., Zhang R., Jin L. J. T. (2019). LncCCAT1 Promotes Breast Cancer Stem Cell Function through Activating WNT/*β*-catenin Signaling. *Theranostics*.

[11] Wang G., Chen H., Liu J. (2015). The long noncoding RNA LINC01207 promotes proliferation of lung adenocarcinoma. *American Journal of Cancer Research*.

[12] Yu S., Li N., Huang Z. (2018). A novel lncRNA, TCONS_00006195, represses hepatocellular carcinoma progression by inhibiting enzymatic activity of ENO1. *Cell Death & Disease*.

[13] Xu S., Kong D., Chen Q., Ping Y., Pang D. (2017). Oncogenic long noncoding RNA landscape in breast cancer. *Molecular Cancer*.

[14] Zhang Y., Sun J., Qi Y. (2020). Long non-coding RNA TPT1-AS1 promotes angiogenesis and metastasis of colorectal cancer through TPT1-AS1/NF90/VEGFA signaling pathway. *Aging*.

[15] Zang X., Gu J., Zhang J. (2020). Exosome-transmitted lncRNA UFC1 promotes non-small-cell lung cancer progression by EZH2-mediated epigenetic silencing of PTEN expression. *Cell Death & Disease*.

[16] Zeitlin S. G. (2010). Centromeres: the wild west of the post-genomic age. *Epigenetics*.

[17] Tomonaga T., Matsushita K., Yamaguchi S. (2003). Overexpression and mistargeting of centromere protein-A in human primary colorectal cancer. *Cancer Research*.

[18] Ambartsumyan G., Gill R. K., Perez S. D. (2010). Centromere protein A dynamics in human pluripotent stem cell self-renewal, differentiation and DNA damage. *Human Molecular Genetics*.

[19] Li Y., Zhu Z., Zhang S. (2011). ShRNA-targeted centromere protein A inhibits hepatocellular carcinoma growth. *PLoS One*.

[20] Wu Q., Qian Y. M., Zhao X. L. (2012). Expression and prognostic significance of centromere protein A in human lung adenocarcinoma. *Lung Cancer*.

[21] Ma X. J., Salunga R., Tuggle J. T. (2003). Gene expression profiles of human breast cancer progression. *Proceedings of the National Academy of Sciences of the United States of America*.

[22] McGovern S. L., Qi Y., Pusztai L., Symmans W. F., Buchholz T. A. (2012). Centromere protein-A, an essential centromere protein, is a prognostic marker for relapse in estrogen receptor-positive breast cancer. *Breast Cancer Research*.

[23] Green M. R., Sambrook J. J. C. S. H. P. (2019). Removing DNA Contamination from RNA Samples by Treatment with RNase-Free DNase I. *Cold Spring Harbor Protocols*.

[24] Nguyen H. P., Daniel P. M., Filiz G., Mantamadiotis T. (2018). Investigating neural stem cell and glioma stem cell self-renewal potential using extreme limiting dilution analysis. *Bio-Protocol*.

[25] Ma F., Song H., Guo B. (2015). MiR-361-5p inhibits colorectal and gastric cancer growth and metastasis by targeting staphylococcal nuclease domain containing-1. *Oncotarget*.

[26] Ma F., Liu X., Zhou S. (2019). Long non-coding RNA FGF13-AS1 inhibits glycolysis and stemness properties of breast cancer cells through FGF13-AS1/IGF2BPs/Myc feedback loop. *Cancer Letters*.

[27] Sanduja S., Blanco F. F., Young L. E., Kaza V., Dixon D. A. (2012). The role of tristetraprolin in cancer and inflammation. *Frontiers in Bioscience: a Journal and Virtual Library*.

[28] Carballo E., Lai W. S., Blackshear P. J. (2000). Evidence that tristetraprolin is a physiological regulator of granulocyte-macrophage colony-stimulating factor messenger RNA deadenylation and stability. *Blood*.

[29] Cao G., Tan B., Wei S. (2020). Down-regulation of MBNL1-AS1 contributes to tumorigenesis of NSCLC via sponging miR-135a-5p. *Biomedicine & Pharmacotherapy*.

[30] Li P., Xing W., Xu J. (2019). microRNA-301b-3p downregulation underlies a novel inhibitory role of long non-coding RNA MBNL1-AS1 in non-small cell lung cancer. *Stem Cell Research & Therapy*.

[31] Wei X., Wang B., Wang Q. (2020). MiR-362-5p, which is regulated by long non-coding RNA MBNL1-AS1, promotes the cell proliferation and tumor growth of bladder cancer by targeting QKI. *Frontiers in Pharmacology*.

[32] Wei X., Yang X., Wang B. (2020). LncRNA MBNL1-AS1 represses cell proliferation and enhances cell apoptosis via targeting miR-135a-5p/PHLPP2/FOXO1 axis in bladder cancer. *Cancer Medicine*.

[33] Zhu K., Wang Y., Liu L., Li S., Yu W. (2020). Long non-coding RNA MBNL1-AS1 regulates proliferation, migration, and invasion of cancer stem cells in colon cancer by interacting with _MYL9_ via sponging microRNA-412-3p. *Clinics and Research in Hepatology and Gastroenterology*.

[34] Xu L., Zhu S., Tang A., Liu W. J. I. R. (2021). LncRNA MBLN1-AS1 inhibits the progression of retinoblastoma through targeting miR-338-5p-Wnt/*β*-catenin signaling pathway. *Inflammation Research*.

[35] Wu J. C., Luo S. Z., Liu T., Lu L. G., Xu M. Y. (2019). linc-SCRG1 accelerates liver fibrosis by decreasing RNA-binding protein tristetraprolin. *Faseb Journal: Official Publication of the Federation of American Societies for Experimental Biology*.

[36] Hua K., Deng X., Hu J. (2020). Long noncoding RNA HOST2, working as a competitive endogenous RNA, promotes STAT3-mediated cell proliferation and migration via decoying of let-7b in triple-negative breast cancer. *Journal of Experimental & Clinical Cancer Research*.

[37] Brennan S. E., Kuwano Y., Alkharouf N., Blackshear P. J., Gorospe M., Wilson G. M. (2009). The mRNA-destabilizing protein tristetraprolin is suppressed in many cancers, altering tumorigenic phenotypes and patient prognosis. *Cancer Research*.

[38] Hitti E., Bakheet T., Al-Souhibani N. (2016). Systematic analysis of AU-rich element expression in cancer reveals common functional clusters regulated by key RNA-binding proteins. *Cancer Research*.

[39] Sanduja S., Kaza V., Dixon D. A. (2009). The mRNA decay factor tristetraprolin (TTP) induces senescence in human papillomavirus-transformed cervical cancer cells by targeting E6-AP ubiquitin ligase. *Aging*.

[40] Canzoneri R., Naipauer J., Stedile M. (2020). Identification of an AP1-ZFP36 regulatory network associated with breast cancer prognosis. *Breast Cancer Prognosis*.

[41] Pan Q. H., Fan Y. H., Wang Y. Z., Li D. M., Hu C. E., Li R. X. (2020). Long noncoding RNA NNT-AS1 functions as an oncogene in breast cancer via repressing ZFP36 expression. *Journal of Biological Regulators and Homeostatic Agents*.

[42] Ding X., Xu X., He X. F., Yuan Y., Huang Y. H. J. B. (2021). Muscleblind-like 1 antisense RNA 1 inhibits cell proliferation, invasion, and migration of prostate cancer by sponging miR-181a-5p and regulating PTEN/PI3K/AKT/mTOR signaling. *Bioengineered*.

[43] Xu D., Wang Y., Wu J. (2021). ECT2 overexpression promotes the polarization of tumor-associated macrophages in hepatocellular carcinoma via the ECT2/PLK1/PTEN pathway. *Cell Death & Disease*.

[44] Kim H. A., Kim K. J., Seo K. H., Lee H. K., Im S. Y. J. F. L. (2012). PTEN/MAPK pathways play a key role in platelet-activating factor-induced experimental pulmonary tumor metastasis. *FEBS Letters*.

[45] Lin Z., Lu Y., Meng Q. (2018). miR372 promotes progression of liver cancer cells by upregulating erbB-2 through enhancement of YB-1. *Molecular Therapy Nucleic Acids*.

[46] Shen B., Li Y., Ye Q., Qin Y. J. C. G. T. (2021). YY1-mediated long non-coding RNA Kcnq1ot1 promotes the tumor progression by regulating PTEN via DNMT1 in triple negative breast cancer. *Cancer Gene Therapy*.

[47] Valla S., Hassan N., Vitale D. L. (2021). Syndecan-1 Depletion Has a Differential Impact on Hyaluronic Acid Metabolism and Tumor Cell Behavior in Luminal and Triple-Negative Breast Cancer Cells. International journal of molecular sciences. *International Journal Of Molecular Sciences*.

[48] Liu C., Wu H., Li Y. (2017). SALL4 suppresses PTEN expression to promote glioma cell proliferation via PI3K/AKT signaling pathway. *Journal of Neuro-Oncology*.

[49] Kaowinn S., Seo E. J., Heo W., Bae J. H., Chung Y. H. J. B., Communications B. R. (2019). Cancer upregulated gene 2 (CUG2), a novel oncogene, promotes stemness-like properties via the NPM1-TGF-*β* signaling axis. *Biochemical and Biophysical Research Communications*.

[50] Iino K., Mitobe Y., Ikeda K. (2020). RNA-binding protein NONO promotes breast cancer proliferation by post- transcriptional regulation of SKP2 and E2F8. *Cancer Science*.

[51] Weinger J. G., Plaisted W. C., Maciejewski S. M., Lanier L. L., Walsh C. M., Lane T. E. J. S. C. (2014). Activating receptor NKG2D targets RAE-1-expressing allogeneic neural precursor cells in a viral model of *multiple sclerosis*. *Stem Cells*.

[52] Wang J., Lou S. S., Wang T. (2019). UBE3A-mediated PTPA ubiquitination and degradation regulate PP2A activity and dendritic spine morphology. *Proceedings of the National Academy of Sciences*.

