# Supplementary material for: LncRNA MBNL1-AS1 Represses Proliferation and Cancer Stem-Like Properties of Breast Cancer through MBNL1-AS1/ZFP36/CENPA Axis
Source: J Oncol. 2022 Apr 26;2022:9999343. doi: 10.1155/2022/9999343 (PMC9064507; doi:10.1155/2022/9999343)
Supplement: Supplementary Materials — Supplementary Figure 1. The HE-stained tissues of BC and normal. Supplementary Figure 2. The function assays of MCF-7 cells transfected with sh-MBNL1-AS1#2. Supplementary Figure 3. The expression levels of ZFP36 in the BC cells. [file 9999343.f1.docx]

**Supplementary Figure 1**


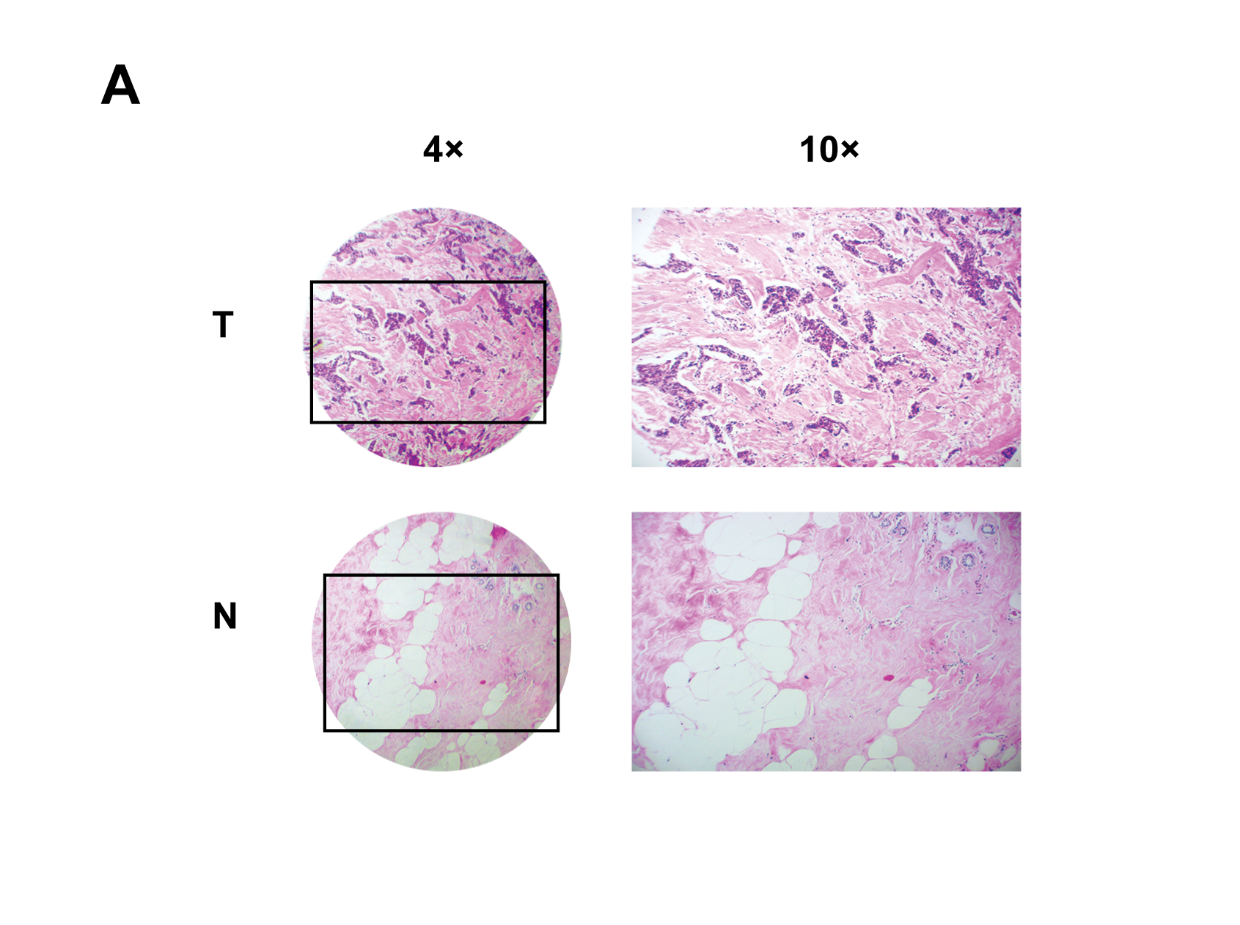


**Supplementary Figure 1** (A)Visualization of the HE-stained tissues from BC and normal

**Supplementary Figure 2**


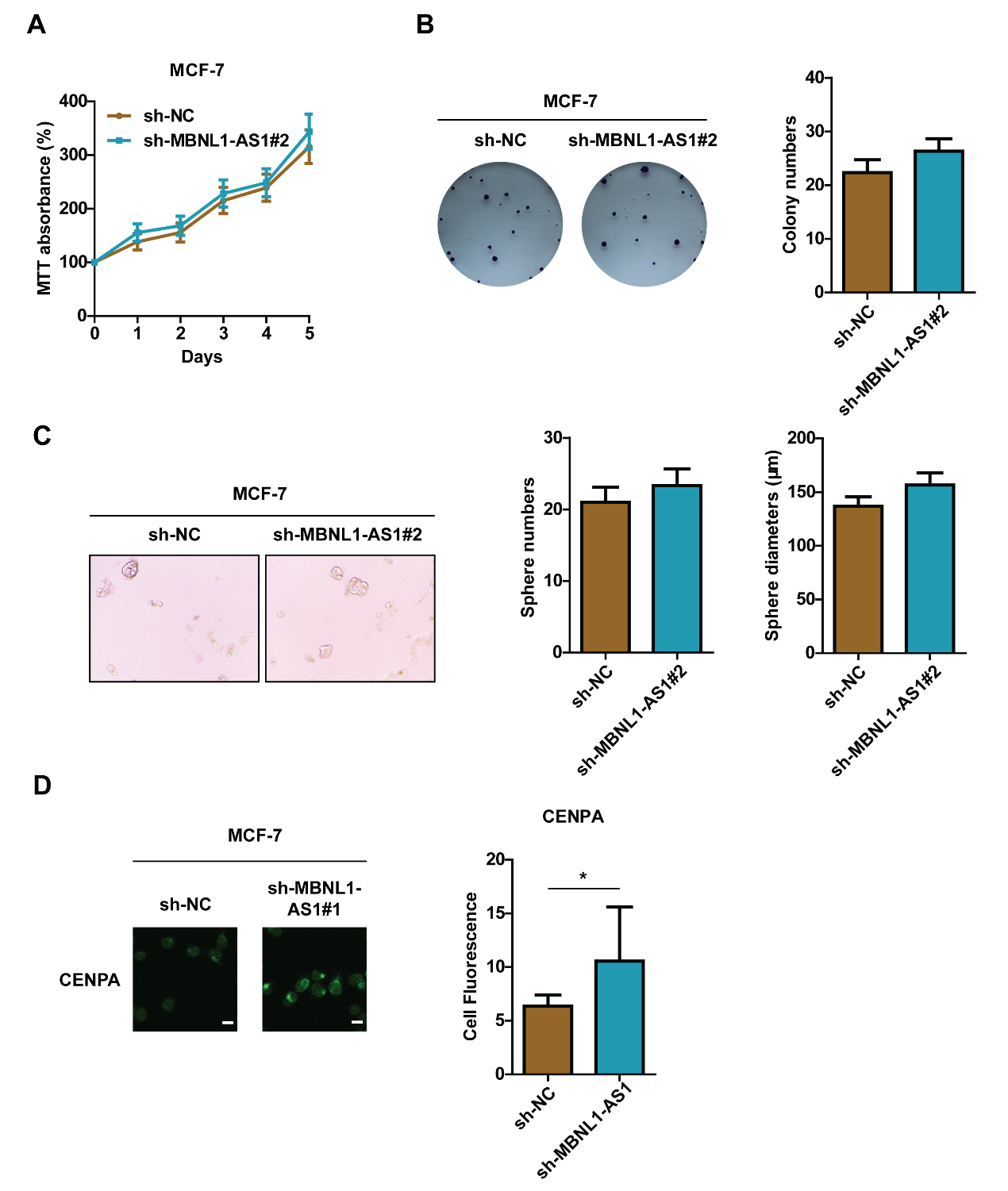


**Supplementary Figure 2** MTT assays (A) and colony formation assays (B) were used to examine the proliferation of MCF-7 cells transfected with sh-MBNL1-AS1#2. (C) Numbers and diameters of tumor sphere in indicated cell. (D) The cell fluorescence of CENPA in MCF-7 cell line

**Supplementary Figure 3**


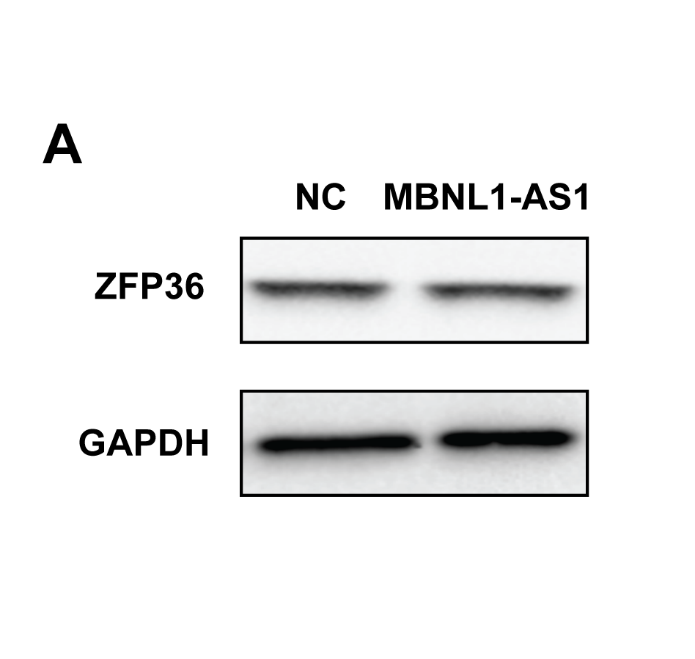


**Supplementary Figure 3** (A) The expression levels of ZFP36 in the BC cells were tested by western blot assays
